# Different formulations of peracetic acid: effects on smear layer removal, dentine erosion, cytotoxicity and antibiofilm activity

**DOI:** 10.1590/1678-7757-2021-0575

**Published:** 2022-03-28

**Authors:** Kennia Scapin Viola, Hernán Coaguila-Llerena, Elisandra Marcia Rodrigues, Cíntia Silva Santos, Gisselle Moraima Chávez-Andrade, Miriam Graziele Magro, Mario Tanomaru, Juliane Maria Guerreiro-Tanomaru, Gisele Faria

**Affiliations:** 1 Universidade Estadual Paulista Faculdade de Odontologia de Araraquara Departamento de Odontologia Restauradora Araraquara SP Brasil Universidade Estadual Paulista - UNESP, Faculdade de Odontologia de Araraquara, Departamento de Odontologia Restauradora, Araraquara, SP, Brasil.

**Keywords:** Cell viability, Dentin, Enterococcus faecalis, Peracetic acid, Smear layer

## Abstract

**Objective:**

To assess the effects of different peracetic acid (PAA) formulations on smear layer (SL) removal, dentine erosion, cytotoxicity, and antibiofilm activity.

**Methodology:**

SL removal and dentine erosion were assessed using 90 premolars, distributed into six groups, according to final irrigation: PAA formulations (1% Sigma, 1% Bacterend OX, 1% Arposept, and 0.09-0.15% Anioxyde), 17% ethylenediaminetetraacetic acid (EDTA), and water (control). Cytotoxicity was assessed by methyl-thiazol-tetrazolium (MTT) and neutral red assays. Antibacterial and antibiofilm effectiveness was evaluated against *Enterococcus faecalis*. For cytotoxicity and antibiofilm activity assessment, the 2.5% NaOCl was also included.

**Results:**

EDTA, Sigma, and Bacterend OX removed more SL than Arposept, Anioxyde, and water (p<0.05). EDTA caused more severe dentine erosion than Sigma and Bacterend OX (p<0.05). Sigma and Bacterend OX had higher cytotoxicity than the other solutions (p<0.05). NaOCl, Bacterend OX, Sigma, and Anioxyde significantly reduced *E. faecalis* colony-forming units (CFU) (p<0.05). The 2.5% NaOCl solution promoted greater biofilm biomass reduction (p<0.05) than the other solutions. All PAA formulations promoted greater biomass reduction than 17% EDTA (p<0.05).

**Conclusions:**

Although Sigma and Bacterend OX had higher cytotoxicity, they had a SL removal capability similar to that of EDTA, were as effective as NaOCl against *E. faecalis* biofilm, and promoted less dentine erosion than EDTA. Arposept and Anioxyde failed to remove the SL, had lower cytotoxicity, and showed less bacterial activity than NaOCl.

## Introduction

Irrigation is an essential step during root canal treatment because areas remain untouched after mechanical instrumentation, allowing bacterial growth.^1^ Although alternative solutions, such as chlorhexidine, octenidine or calcium hypochlorite have been proposed for root canal irrigation;^2,3^ sodium hypochlorite (NaOCl) is the most commonly recommended solution because of its high antimicrobial and antibiofilm activity,^4,5^ and its organic dissolution ability.^6^ However, NaOCl has no effect on inorganic tissue, and requires a chelating agent, such as ethylenediaminetetraacetic acid (EDTA), to remove the smear layer (SL).^2,7-9^ Some irrigation protocols include irrigation with NaOCl after EDTA to optimize disinfection,^10^ since EDTA has low antimicrobial activity and is ineffective against dental biofilm.^5^ Although this method is suitable from a microbiological point of view, the use of NaOCl after EDTA has been found to cause dentine erosion^11^ and reduce the microhardness of root canal dentine.^12^

Using 1% peracetic acid (PAA) as a final irrigating solution has been proposed since it has antibacterial activity similar to EDTA + 2.5% NaOCl in root canals inoculated with *Enterococcus faecalis*,^13^ and an SL removal capability similar to 17% EDTA.^14^ PAA can be produced via three pathways. The first is known as the classical or conventional pathway and consists of obtaining PAA by a chemical reaction between hydrogen peroxide and acetic acid using a catalyst, resulting in PAA and water. Sulfuric acid is usually used as the catalyst. This kind of PAA is available as an aqueous solution, in which PAA is formed in an equilibrium mixture with hydrogen peroxide, acetic acid, and water.^15^ Specific concentrations or types of PAA are obtained by regulating the concentration of hydrogen peroxide and acetic acid during the manufacturing process. The PAA produced via the classical pathway is the most commonly used form for disinfection and removal of the SL in the root canal system.^4,13^

The second method is called the alternative pathway and is used to obtain the PAA produced by the PHERA^®^ system (Laboratoires Anios, Lille-Hellemmes, France). A reaction occurs when 3% hydrogen peroxide (generator solution) is mixed with an activating solution containing acetyl caprolactam. The final product contains no acetic acid.^16^

In the third pathway, PAA is formed *in situ* by dissolving a powdered product containing an activator (tetraacetylethylenediamine - TAED) and a persalt (sodium percarbonate or sodium perborate) in water.^15^ PAA solutions formulated *in situ* also contain hydrogen peroxide, but no acetic acid.^17^

According to hypotheses in the literature, the acetic acid in the PAA produced via the classical pathway would be responsible for SL removal.^14^ However, acetic acid is a weak acid,^18^ lacking a concentration of H^+^ ions that could provide efficient calcium and SL removal.^19^ On the other hand, considering the chemical structure of the PAA molecule, it could have chelating action due to the presence of two coordination sites, namely carbonyl (C=O) and peroxide groups (-O-O-), which can form complexes with calcium.^20^ Therefore, it is important to evaluate whether the PAA produced by PHERA^®^ and *in situ* pathways are able to remove the SL. Additionally, it is important to assess dentine erosion, antimicrobial activity, and cytotoxicity because they are factors, among others, that are considered for an “ideal” irrigating solution.^21^ Comparing different PAA formulations, those aspects have not yet been investigated.

The aim of this study was to assess the effects of PAA solutions produced by different pathways on SL and dentine erosion, as well as the cytotoxicity and antibacterial/antibiofilm activity of these solutions. The null hypothesis was that there would be no differences among solutions regarding effects on SL removal, dentine erosion, cytotoxicity, and antibiofilm activity.

## Methodology

The sample size required to perform each assay was estimated using the G* Power 3.1 software program for Windows. SL evaluation by scores was achieved by estimating sample size based on an effect size = 0.8 (obtained from the pilot study), a test power (β) =0.95, and α=0.05, using the “F-test family” for one-way analysis. Estimation showed that 42 specimens (7 per group) would be required. Erosion evaluation by scores was achieved by estimating sample size based on an effect size =0.85 (obtained from the pilot study), test power (β) =0.95, and α=0.05, using the “F-test family” for one-way analysis. Estimation showed that 36 specimens (6 per group) would be required. Since score data are assessed by non-parametric tests, 15% of the specimens were added, as recommended in the literature.^22,23^ Additionally, 20% of the losses that may occur during the experiment were added, resulting in the following sample size: 60 specimens for SL and 54 specimens for erosion. The sample size estimate for the open dentinal tubule analysis showed that 7 specimens per group were needed (effect size =0.80, obtained from a pilot study; test power [β] =0.85; and α=0.05, using the “F-test family” for one-way analysis). However, we used 90 specimens (n=15 per group) based on the estimate of the sample size needed to analyze the number of open tubules (effect size =0.52, obtained from a pilot study; test power [β] =0.85; and α=0.05, using the “F-test family” for one-way analysis).

### Preparation of irrigating solutions

PAA solutions were prepared immediately before use by diluting a commercial product (Table 1) in deionised water. EDTA at a concentration of 17% (Biodinâmica, Ibiporã, PR, Brazil), 2.5% NaOCl, and distilled water were used as controls. The NaOCl solution was titrated using physicochemical spectrophotometry to determine the free available chlorine. NaOCl at 2.5% was prepared immediately before use by diluting a 9% NaOCl solution in distilled water.

**Table 1:** Types of peracetic acids, manufacturers, composition, and concentrations used in the irrigating solution

Peracetic Acid (commercial product)	Pathway	Main feature	Composition			Concentration used as irrigating solution		
			PAA	H_2_O_2_	AA	PAA	H_2_O_2_	AA
Peroxyacetic acid –Sigma (Sigma-Aldrich, St. Louis, MO, USA)	Classical [H_2_0_2_+AA]	With AA Low % of H_2_0_2_	36-40%	5-8%	40%	1%	0.125-0.2%	1%
Bacterend OX (Profilática, Curitiba, PR, Brazil)	Classical [H_2_0_2_+AA]	With AA High % of H_2_0_2_	4%	26%	NA	1%	6,5%	NA
Arposept (ARPO Chimie et Technologie Sàrl, Villaz-St-Pierre, Glâne, Fribourg, Switzerland)	*In situ* [activator (TAED) + persalt + water]	Without AA	0	0	0	1%	NA	0
Anioxyde 1000 (Laboratoires Anios, Lille-Hellemmes, France)	PHERA® [3% H_2_0_2_+activator (acetyl caprolactam)]	Without AA	0.09- 0.15%	3%	0	0.09- 0.15%	3%	0

PAA: peracetic acid, H_2_O_2_: hydrogen peroxide, AA: acetic acid, NA: concentration not available

### Assessment of smear layer removal and dentine erosion

After approval by the Ethics Committee of the School of Dentistry (CAAE: 08838019.7.0000.5416), 90 single-rooted human mandibular premolars with similar dimensions, single round-shaped canals, straight roots (< 5° Schneider angle), and single foramen (Vertucci type I configuration) were used. Specimen selection was performed using a stereomicroscope and radiographs. Radiographs were taken using a digital sensor and analysed using the Image J software program (National Institutes of Health, Bethesda, MD, USA) to confirm inclusion criteria^24^. Round-shaped canals were considered when the buccolingual diameter equalled the mesiodistal diameter.^25^ Specimens were randomly allocated into six experimental groups (n=15).

Crowns were removed and root lengths were standardized at 16 mm. Working length was established at one mm short of the apical foramen, and the foraminal opening was sealed using composite resin. Root canals were instrumented by the same operator up to the F5 file of the ProTaper Universal rotary system (Dentsply Sirona Endodontics, Ballaigues, Switzerland), following the technique recommended by the manufacturer. Root canals were irrigated with 2.5 mL of 2.5% NaOCl for one min at each change of file.

**Figure 1:**
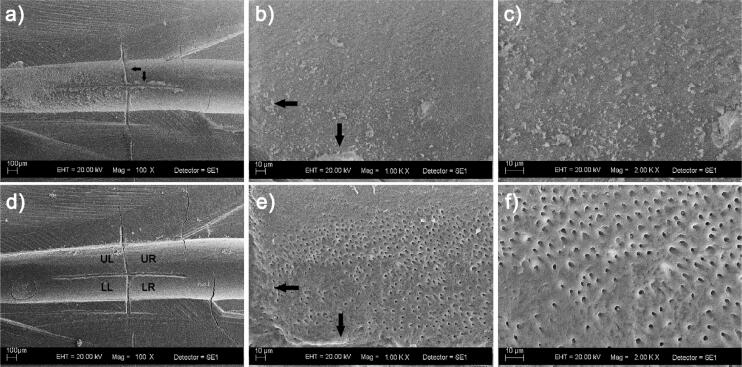
Marking made on the region to evaluate the smear layer on root canal dentine. A cross-shaped mark was made on the apical segment (black arrows) to standardize the same pre- (a,b,c) and post-final irrigation areas (d,e,f). The original magnifications of the marked areas are 100X (a,d), 1000X (b,e), and 2000X (c,f). LL: lower left area; LR: lower right area; UL: upper left area; UR: upper right area

After chemo-mechanical preparation, but before final irrigation, roots were prepared following the methodology used by Schmidt, et al.^26^ (2015), but with modifications. Two parallel grooves were made on the buccal and lingual surfaces of the roots, using a 0.08 mm diamond disc (Discoflex, KG Sorensen, Cotia, SP, Brazil) at low speed, making sure to avoid contact with the canal. Condensation silicone (Zetaplus, Zhermack, Badia Polesine, RO, Italy) was placed in a two mL microtube with its cap cut off, and roots were embedded in it up to the level of the cemento-enamel junction. After the silicone set, roots were cleaved with a chisel to produce two halves. One of the halves was used for pre-irrigation assessment using a scanning electron microscope (SEM) (EVO 50, Carl Zeiss, Oberkochen, Germany). Two perpendicular markings were made in the root canals with a #12 scalpel blade at 3 (apical segment) and 7 mm (middle segment) from the apex. These markings allowed a cross-shaped image to be observed in each segment for SEM analysis. Halves were kept in an incubator for three days at 37ºC for dehydration. They were then evaluated using SEM at low vacuum with no metallisation/additional preparation. After the markings in the canal (Figure 1) were located, images of each segment were obtained at 100X and 1000X to determine the same areas to be assessed before and after final irrigation, and at 2000X, to confirm the SL formation before final irrigation. Then, the two halves were placed together in the microtubes containing the silicone matrix to proceed with the final irrigation protocols. Specimens were distributed into six groups (n=15) according to the final irrigating solution: 1% Bacterend OX, 1% Sigma, 1% Arposept, 0.09 to 0.15% Anioxyde, 17% EDTA, and distilled water. The final irrigation volume was 3 mL for 3 min, after which specimens were irrigated with 5 mL of distilled water for 2 min to prevent any residual effects of the solutions on the dentine. A 5 mL disposable plastic syringe (Ultradent, South Jordan, UT, USA), coupled to a 27G side-vented needle (Endo-EZE, Ultradent), was used in the irrigation process. The needle was placed 1 mm short of the working length. Halves were separated, dehydrated, and then examined by SEM at 10 kV. Smear layers were assessed by obtaining images of the predetermined areas in the middle and apical segments in the pre-irrigation SEM images.

The scores proposed by Hülsmann, Rümmelin, and Schäfers^27^ (1997) for SL evaluation were used in the images acquired at 2000X. The Image J software program (National Institutes of Health, NIH) was used to measure the area^26^ and to count the open dentinal tubules^28^ in the images acquired at 5000X, which were taken from the same region used for SL evaluation and corresponded to a 2241 μm^2^ area. The increase in magnification to 5000X was performed without any alteration to specimen position. Also, the previously selected areas for SL removal assessment were used for dentine erosion evaluation by analyzing SEM images at 5000X, using the score criteria developed by Torabinejad, et al.^7^ (2003). Two calibrated, blinded examiners attributed scores to smear layers and to erosion, and then counted and measured the open dentinal tubules [intra-class correlation coefficient (ICC) =0.99 for tubule count and ICC =0.96 for area measurement].

### Cytotoxicity evaluation by methyl-thiazol-tetrazolium (MTT) and neutral red assays

After this study was approved by the Ethics Committee of the School of Dentistry and consent forms were signed, three young and healthy patients aged 16-25 years from the oral surgery department were recruited. Periodontal ligaments (hPDL) were collected from extracted human third molars without caries or periodontal disease.

After expansion of the hPDL cell cultures, cells (8x10^4^ cells/mL) were cultured in 96-well culture plates (Corning, NY, USA) using Dulbecco’s Modified Eagle Medium (DMEM), supplemented with 10% foetal bovine serum (FBS), and incubated for 24h to promote adherence to the plates. Then, the culture medium was removed and cells were exposed to irrigating solutions for 3 min. The solutions described in Table 1, plus 17% EDTA and 2.5% NaOCl, were considered grade 1 dilutions,^3,29^ and were subjected to serial dilutions in a saline solution (sodium chloride 0.9%) using a dilution factor of 1.5. Cells were exposed to irrigant solutions at 0.0002% to 0.01% doses/concentrations, previously selected from pilot studies to obtain a dose-response curve. Saline and DMEM were used as controls. Then, irrigants were removed, and cells were incubated in DMEM supplemented with 10% FBS for 4h,^3,29^ after which the cytotoxicity tests were performed.

The MTT assay was performed by using an MTT solution (Sigma-Aldrich) at 0.5 mg/mL. The neutral red assay was performed by using a neutral red solution (Sigma-Aldrich) at 0.05 mg/mL. The optical densities for both assays were measured with a spectrophotometer at 570 nm. The percentage of cell viability was estimated from the absorbance of the control (saline), considered as 100%. The experiments were performed in triplicate and repeated three different times.

### Antimicrobial and antibiofilm activity against *E. faecalis*

#### Direct contact test on bovine dentine blocks

Bovine dentine blocks (n=42) measuring 5 mm x 5 mm x 0.7 mm (width x length x thickness) were immersed in an *E. faecalis* (ATCC 29212) suspension (1x10^7^ CFU/mL). Gram staining and colony morphology were performed to confirm the purity of the strain. The microorganism was reactivated in 4 mL of Brain Heart Infusion (BHI) agar broth and kept at 37°C for 12 hours. Each block was placed in a 24-well plate so the surface marked with graphite faced downwards. Then, the blocks were immersed in 1.8 mL of the BHI medium and 200 μL of the *E. faecalis* suspension, adjusted in a spectrophotometer at DO600 =0.060 and equivalent to 1x10^7^ colony-forming units per mL (CFU/mL). The plates were incubated for 15 days at 37ºC on a rocking table, maintaining microaerophilic conditions. The BHI medium for each specimen was renewed every 48 hours.*E*.*faecalis* biofilm formation was confirmed by gram staining and colony morphology. The blocks were distributed into groups according to Table 1, plus 17% EDTA, 0.85% saline solution (negative control), and 2.5% NaOCl (positive control). The blocks were immersed in 1 mL of each solution for 3 min. Afterwards, they were washed in saline and placed in microtubes containing a neutralizing solution (5% sodium thiosulfate) for 5 min. The microtubes were shaken for 60 sec to disrupt the biofilm. Aliquots of each dilution were inoculated on Petri plates containing TSa (Difco Detroit, MI, USA), which were incubated at 37°C for 24h. Results were obtained by estimating the mean number of CFUs in the three bacterial growth areas in the dilution. Averages were submitted to log transformation.^30^

#### Crystal violet assay

This assay was performed to assess the effectiveness of solutions against *E. faecalis* biofilm biomass.^30^ *E. faecalis* was plated in 96-well microtiter plates (NEST Biotechnology, Wuxi, China) in the culture medium, at 37°C for 48h to allow biofilm formation. An aliquot of 200 μL of each solution was then added for 3 min. The culture medium with the standard inoculum (positive control) and the sterile culture medium (negative control) were used as controls. After removing the solutions, the wells with biofilm were stained with 0.1% crystal violet solution (Synth, Diadema, SP, Brazil) for 20 min. The plates were dried, and the dye, attached to adherent cells, was solubilized with 33% acetic acid for 5 min. The quantification of the remaining biofilm biomass was performed in a spectrophotometer (590 nm). The reduction in biofilm biomass was estimated as a percentage (%) of the positive control.^30^ This assay was performed in triplicate and repeated three different times.

### Statistical analysis

Data were analyzed using SPSS version 20 (IBM Corp, Armonk, NY, USA) and Graph Pad Prism 5 (GraphPad Software, La Jolla, CA, USA) statistical software programs (α=0.05). The intra-class correlation coefficient (ICC) was used to evaluate inter-examiner concordance in counting and measuring open dentinal tubules. Since the scores of SL and dentine erosion had non-normal distributions, they were analyzed using nonparametric Kruskal-Wallis and Dunn post-test (comparison between groups) or Wilcoxon tests (comparison between segments). Other results were analyzed using one-way ANOVA and Tukey’s post-test (crystal violet assay and direct contact test), as well as two-way ANOVA and the Bonferroni post-test (cytotoxicity, and number and area of open dentinal tubules), because the normal distribution of data was confirmed in a preliminary analysis using the D’Agostino-Pearson normality test.

## Results

### Smear layer removal and dentine erosion

**Figure 2:**
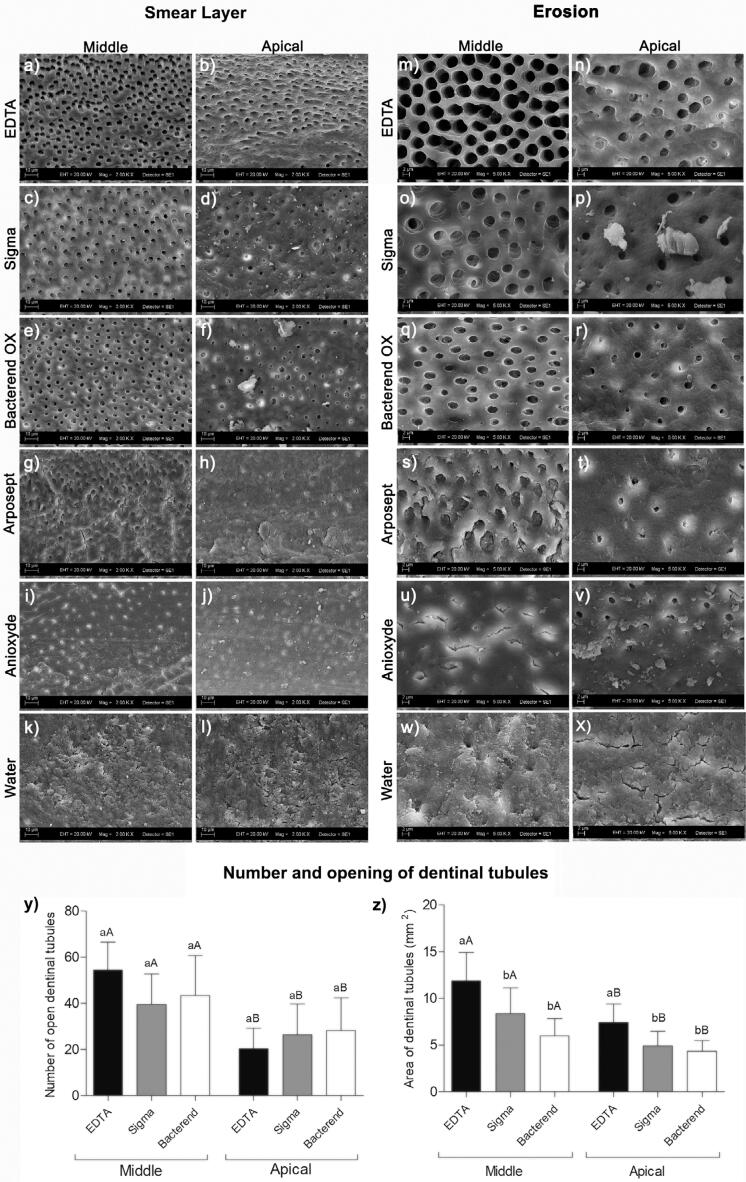
Effects on root canal dentine. Representative SEM images of smear layers in the middle and apical segments after final irrigation with EDTA (a,b), Sigma (c,d), Bacterend OX (e,f), Arposept (g,h), Anioxyde (i,j), and distilled water (k,l) groups. Bar = 10μm. Representative SEM images of erosion in the middle and apical segments after final irrigation with EDTA (m,n), Sigma (o,p), and Bacterend OX (q,r). Images of Arposept (s,t), Anioxyde (u,v), and distilled water (w,x) groups correspond to the specimens with the minimum score obtained in assessing smear layer removal. Bar = 2μm. Number (y) and opening of dentinal tubules (z) in the middle and apical segments after final irrigation with EDTA, Sigma, and Bacterend OX. Different lowercase letters indicate significant differences among solutions in the same segment. Different uppercase letters indicate significant differences between each segment, considering the same solution

EDTA, Sigma, and Bacterend OX promoted higher SL removal rates than Arposept, Anioxyde, and distilled water (p<0.05). Comparison between the segments showed that EDTA, Sigma, and Bacterend OX promoted lower SL removal rates in the apical segment than in the middle one (p<0.05). Arposept, Anioxyde and distilled water showed no significant differences among the segments (p>0.05) (Table 2 and Figure 2).

**Table 2:** Median, minimum, maximum, and first and third quartiles of smear layer and erosion scores after final irrigation with different PAA solutions, EDTA, and distilled water

SMEAR LAYER					
Segment	Group	Median	Minimum	Maximum	1^st^-3^rd^ Quartiles
Middle	EDTA	1^aA^	1	2	1 – 2
	Sigma	1^aA^	1	3	1 – 1.25
	Bacterend OX	1^aA^	1	2	1 – 1
	Arposept	3.5^bA^	2	5	3 – 5
	Anioxyde	4^bA^	3	5	4 – 4
	Water	4^bA^	3	5	3 – 5
Apical	EDTA	2^aB^	1	3	1 – 3
	Sigma	2^aB^	1	3	1 – 2.5
	Bacterend OX	2^aB^	1	4	1 – 2.75
	Arposept	4^bA^	3	5	3 – 5
	Anioxyde	4^bA^	3	5	3.75 – 5
	Water	5^bA^	4	5	4 – 5

EROSION					
Segment	Group	Median	Minimum	Maximum	1^st^-3^rd^ Quartiles
Middle	EDTA	3^bB^	3	3	3 – 3
	Sigma	2^aB^	1	3	1.75 – 2
	Bacterend OX	1^aB^	1	3	1 – 2
Apical	EDTA	1.5^aA^	1	2	1 – 2
	Sigma	1^aA^	1	3	1 – 1.25
	Bacterend OX	1^aA^	1	2	1 – 1

Different lowercase letters in each segment indicate a significant difference among the solutions (p<0.05). Different capital letters indicate a significant difference between segments for each solution (p<0.05)

Arposept, Anioxyde, and distilled water were excluded from the erosion assessment since they failed to remove the SL. However, EDTA promoted more severe dentine erosion than Sigma and Bacterend OX in the middle segment (p < 0.05) (Table 2 and Figure 2). There was no difference in the number of tubules among EDTA, Sigma, and Bacterend OX in either middle or apical segments (p>0.05). EDTA, Sigma, and Bacterend OX produced more open dentinal tubules in the middle than in the apical segment (p<0.05) (Figure 2). Furthermore, EDTA, Sigma, and Bacterend OX produced a greater opening of dentinal tubules in the middle than in the apical segment (p<0.05). Interestingly, Bacterend OX and Sigma produced a smaller opening of dentinal tubules than EDTA in both segments (p<0.05) (Figure 2).

### Cytotoxicity

**Figure 3:**
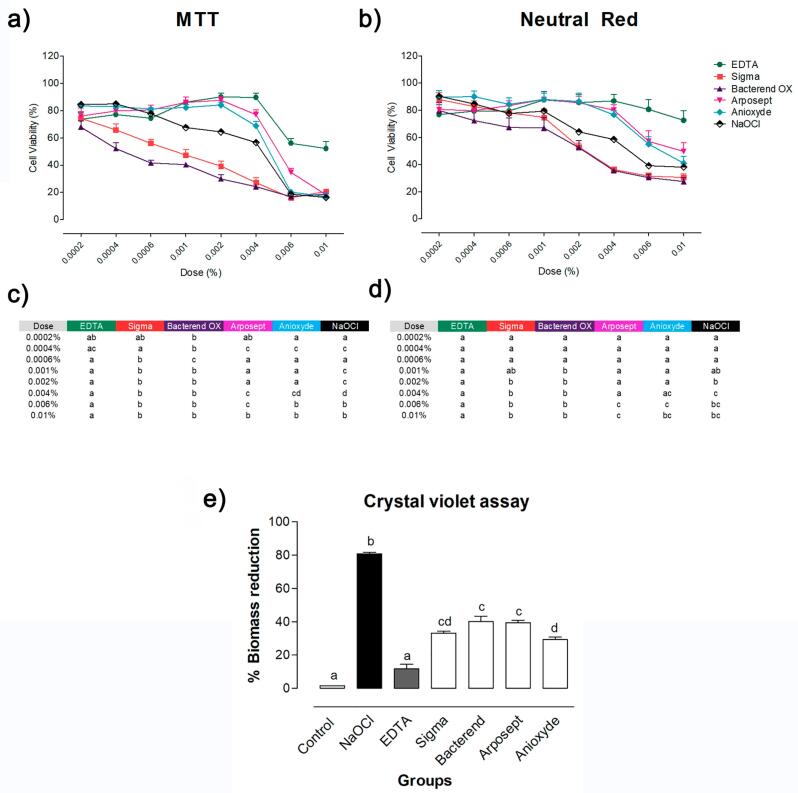
Cytotoxicity and antimicrobial activity. Viability of periodontal ligament cells after exposure to solutions tested at different doses by MTT (a) and neutral red (b) assays. Statistical comparison of MTT (c) and neutral red (d) results. Different letters in rows indicate significant differences among the solutions (p < 0.05) for each dose. Crystal violet assay (e). Percentage of biomass reduction in the *E. faecalis* biofilm. Different letters indicate significant differences among the groups (p < 0.05)

There was no difference between Sigma and Bacterend OX in regard to cytotoxicity, but both had higher rates than the other solutions (p<0.05). EDTA had lower cytotoxicity than the other solutions at higher doses (p<0.05). There was no difference between Anioxyde and Arposept (p>0.05) (Figure 3).

### Antimicrobial and antibiofilm activity against *E. faecalis*

In the direct contact test, there was no difference between EDTA and saline solution (p>0.05). NaOCl, Bacterend OX, Sigma, and Anioxyde promoted a reduction of more than five logarithmic units of *E. faecalis* CFU/mL, compared to the untreated control (p<0.05). There was no difference among NaOCl, Bacterend OX, and Sigma (p<0.05). Arposept had lower antibacterial activity than the other PAA formulations (p<0.05). However, it had higher activity than EDTA (p<0.05) (Table 3). The 17% EDTA group had the lowest biomass reduction and was significantly indifferent from the control (saline) (p>0.05). All PAA formulations promoted higher biomass reduction than the control and 17% EDTA (p<0.05), but lower than NaOCl (p<0.05) (Figure 3).

**Table 3:** Mean and standard deviation of the mean obtained in CFU/mL log10 of *E. faecalis*

Group	CFU/mL log10
Saline (control)	8.44 (±0.13)^a^
NaOCl	0.0 (±0.0)^b^
EDTA	7.48 (±0.08)^a^
Sigma	1.68 (±1.89)^bc^
Bacterend OX	0.0 (±0.0)^b^
Arposept	5.28 (±0.46)^d^
Anioxyde	1.97 (±1.60)^c^

Different letters indicate significant differences among the groups (p < 0.05).

## Discussion

The null hypothesis was rejected since the results showed differences among the PAA formulations and EDTA.

The irrigation time used with the final irrigants was 3 min for two reasons: first, this is the reported time for PAA antimicrobial activity against *E. faecalis*;^13^ second, the dentine erosion caused by EDTA is similar whether applied for 1 or 3 min, as long as there is no irrigation with NaOCl after using EDTA.^11^ However, it has also been reported that 1-min EDTA irrigation is effective in removing the smear layer with no dentine erosion.^31^ Middle and apical segments were used to evaluate PAA effects on dentine, as previously reported.^28^

The conventional SEM analysis for evaluating SL removal has limitations related to a non-longitudinal and non-three-dimensional analysis.^32^ In this study, markings were performed on the dentine to allow the same areas to be analyzed before and after final irrigation.^26^ This longitudinal analysis prevents areas not touched by instrumentation from being erroneously scored as SL-free areas and also prevents the selection of the observation area after final irrigation from being operator-dependent, factors considered limitations of conventional SEM analyses.^32^

The inorganic part of the SL must be removed with a chelating or acid solution.^2,14^ We hypothesize that the chemical structure of the PAA molecule can provide it with the chelating action needed to promote removal of the SL. However, the PAA formulations of PHERA^®^ and *in situ* pathways (Anioxyde and Arposept, respectively) were ineffective in removing the SL. Speculatively, this ineffectiveness may be also attributed to their concentrations. Both solutions may require a higher concentration to exert a chelating effect, as previously demonstrated for EDTA.^33^ It is important to consider that the PAAs tested in this study are industrial/commercial products with potential repurpose for endodontic irrigation.

The PAA formulations for the classical pathway (Sigma and Bacterend OX, pH < 3) promoted an SL removal similar to EDTA, corroborating the results of previous studies.^8,9,14^ The Sigma solution used here had 1% PAA, 0.2% hydrogen peroxide, and 1% acetic acid in its composition, whereas Bacterend OX had 1% PAA, 6.5% hydrogen peroxide, and acetic acid in an amount not revealed by the manufacturer. The SL removed by PAA using the classical pathway has been hypothetically attributed to the acetic acid in the composition of PAA solutions produced by the classical pathway.^8,14^ However, the 1% acetic acid present in the Sigma composition would probably be insufficient to remove the SL in a manner similar to that of EDTA. This can be explained because 5% acetic acid is significantly less effective than 17% EDTA in removing the SL and calcium ions from the root canal.^19^ Additionally, acetic acid is a weak acid,^18^ and lacks a concentration of H^+^ ions that could effect efficient calcium removal,^19^ especially at low concentrations such as 1%. A possible explanation for the removal of the SL by Sigma and Bacterend OX could involve their additives, such as the sulfuric acid added during the manufacturing process to accelerate the establishment of the final equilibrium concentration of PAA from the classical pathway.^15^ However, more research is needed to confirm this hypothesis.

The dentine treated with EDTA produced a greater opening of dentinal tubules and greater dentine erosion than the dentine treated with Sigma or Bacterend OX. These two effects promoted by EDTA could be attributed to the severe demineralization it produces versus the slight demineralization brought about by the PAA produced by the classical pathway.^34^ Our dentine erosion results are in accordance with a study^35^ that used Bacterend OX (Peresal) in the same concentration as the root canal irrigant. Arposept, Anioxyde, and water failed to promote the removal of the SL, thus precluding dentine erosion assessment.

EDTA had lower cytotoxicity in hPDL cells than NaOCl and all PAA formulations. Previous studies^36-38^ have reported that 17% EDTA was less cytotoxic than NaOCl in different cell lines. Arposept and Anioxyde promoted a cytotoxic level close to that of EDTA, whereas Sigma and Bacterend OX promoted higher cytotoxicity than Arposept and Anioxyde. In this regard, the close-to-neutral pH of Arposept (7.5-8.5) and Anioxyde (5.5-7), and the acidic pH, from the acetic acid, of Sigma and Bacterend OX (< 3) may have played a critical role since an acidic pH promotes an unfavourable condition for cells.^39,40^ In summary, cytotoxicity was associated to lower pH levels and the PAA solutions that achieved higher SL removal were the most cytotoxic.

The 0.0002% to 0.01% doses/concentrations were determined in pilot tests to assess the dose-response curve, which is important because a high concentration is not always highly cytotoxic, and low concentrations can also cause high cytotoxic effects.^41^ It is important to highlight that irrigants were diluted in saline and placed in contact with the hPDL cells for 3 min. They were not diluted in a culture medium because it contains buffering substances that neutralize both the acidic pH of PAA produced by the classical pathway and the alkaline pH of NaOCl. Neutralization of irrigating solutions is undesired because it alters their conditions for clinical use and may invalidate results.^29^ The higher cytotoxicity promoted by Sigma, in comparison to NaOCl, agrees with a previous study^29^ that used L929 fibroblasts.

In the crystal violet assay, NaOCl promoted the highest reduction in the biofilm biomass of *E. faecalis*, as expected, because of its biofilm dissolution capability.^30^ However, since the crystal violet assay stains the extracellular matrix, as well as viable and dead cells,^42^ we performed an *E. faecalis* CFU count. NaOCl and all PAA formulations were more effective than EDTA in reducing *E. faecalis* viability in biofilm. The ineffective antibiofilm action of EDTA^5^ and the high antibiofilm action of NaOCl and PAA^4^ have been previously reported. Studies have reported that 1% PAA, 2.5% NaOCl, and 17% EDTA + 2.5% NaOCl induced a similar reduction in *E. faecalis*.^4,13^ Sigma and Bacterend OX had an effect similar to that of NaOCl, but Arposept and Anioxyde had lower activity against *E. faecalis* viability in biofilm. This can be explained by the 3-min contact time of the latter pair with the biofilm used in this study. According to the manufacturer, Anioxyde has bactericidal, fungicidal, virucidal, and sporicidal activity at 5 min of contact.^16^ The manufacturer of Arposept recommends a concentration of 1-2% for 15 min to obtain high-level disinfection of instruments.^43^ It is important to consider that the root canal is more complex in structure than dentine blocks. Additionally, the biofilm formed on a culture plate is different to that formed under clinical conditions.

Finally, it is well established that clinicians must consider several parameters when choosing an irrigating solution.^21^ Based on the observations of this study, the PAAs produced by the classical pathway promoted favourable antibacterial activity and effects on dentine. However, they were more cytotoxic than the other solutions. Therefore, clinicians must assess risk/benefit when choosing this type of irrigating solution, especially in teeth with an open apex, in which the irrigant interacts with cells of the periradicular region, besides having a higher possibility of extrusion.

## Conclusion

Although PAA formulations of the classical pathway (Sigma and Bacterend OX) had higher cytotoxicity, they had a smear layer removal capability similar to that of EDTA, were as effective as NaOCl against *E. faecalis* biofilm, and promoted lower dentine erosion than EDTA. Arposept and Anioxyde did not remove the smear layer and had lower cytotoxicity and lower bacterial activity than NaOCl.
